# Establishment and application of a VP3 antigenic domain-based peptide ELISA for the detection of antibody against goose plague virus infection

**DOI:** 10.3389/fmicb.2023.1309807

**Published:** 2023-11-23

**Authors:** Dandan Qiao, Lainlian Wu, Chenxi Gu, Hongxia Shao, Yongxiu Yao, Aijian Qin, Ankang Hu, Kun Qian

**Affiliations:** ^1^Laboratory Animal Center, Xuzhou Medical University, Xuzhou, China; ^2^Ministry of Education Key Laboratory for Avian Preventive Medicine, Yangzhou University, Yangzhou, China; ^3^Jiangsu Key Laboratory of Preventive Veterinary Medicine, Yangzhou University, Yangzhou, China; ^4^Jiangsu Co-innovation Center for Prevention and Control of Important Animal Infectious Diseases and Zoonoses, Yangzhou University, Yangzhou, Jiangsu, China; ^5^The Pirbright Institute & UK-China Centre of Excellence for Research on Avian Diseases, Woking, United Kingdom

**Keywords:** goose parvovirus, antigen region alignment analysis, peptide ELISA, goose serum, antibody detection

## Abstract

The detection of antibody against goose plague virus (GPV) infection has never had a commercialized test kit, which has posed challenges to the prevention and control of this disease. In this study, bioinformatics software was used to analyze and predict the dominant antigenic regions of the main protective antigen VP3 of GPV. Three segments of bovine serum albumin (BSA) vector-coupled peptides were synthesized as ELISA coating antigens. Experimental results showed that the VP3-1 (358-392aa) peptide had the best reactivity and specificity. By using the BSA-VP3-1 peptide, a detection method for antibody against GPV infection was established, demonstrating excellent specificity with no cross-reactivity with common infectious goose pathogen antibodies. The intra-batch coefficient of variation and inter-batch coefficient of variation were both less than 7%, indicating good stability and repeatability. The dynamic antibody detection results of gosling vaccines and the testing of 120 clinical immune goose serum samples collectively demonstrate that BSA-VP3-1 peptide ELISA can be used to detect antibody against GPV in the immunized goose population and has higher sensitivity than traditional agar gel precipitation methods. Taken together, the developed peptide-ELISA based on VP3 358-392aa could be useful in laboratory viral diagnosis, routine surveillance in goose farms. The main application of the peptide-ELISA is to monitor the antibody level and vaccine efficacy for GPV, which will help the prevention and control of gosling plague.

## 1 Introduction

Since Fang (1962) first discovered and reported goose plague in Yangzhou in 1956, cases of goose plague have also been reported in various countries around the world (Swayne, 2020). Goose plague is a highly contagious disease caused by Goose Parvovirus (GPV) infection, primarily affecting goslings and ducklings under 1 month of age. The mortality rate approaches 100% after infection with the virus (Calnek, 1991; Swayne, 2020). Goose plague significantly impacts the development of the goose farming industry world widely, which causing substantial economic losses in well-established goose farming regions.

Goose Parvovirus (GPV) belongs to the family *Parvoviridae* and the *Dependovirus* genus. Its genome is a single-stranded linear DNA with a size of approximately 5,106 nt. GPV primarily encodes three structural proteins: VP1, VP2, and VP3, as well as two non-structural proteins: NS1 and NS2 (Zádori et al., 1994; Brown et al., 1995; Stefancsik et al., 1995). VP1, VP2, and VP3, these three proteins share a single open reading frame with the same termination codon. VP2 and VP3 are translated internally from VP1, which meaning that VP1 contains all the amino acid sequences for VP2 and VP3 (Stefancsik et al., 1995; Tsai et al., 2004). The structural protein VP3 is the primary capsid protein, constituting approximately 80% of the total capsid proteins. It contains the major immunoprotective antigens on the surface of GPV viral particles, capable of inducing the production of neutralizing antibodies in animals. Moreover, it exhibits relatively low variability, making it an ideal antigen for serological detection of GPV (Ju et al., 2011; Swayne, 2020).

Currently, methods that have been reported for detecting GPV antibodies include agar gel precipitation, neutralization tests, indirect immunofluorescence, immunoblotting, and ELISA (Kisary, 1985; Kardi and Szegletes, 1996; Takehara et al., 1999; Wang et al., 2005; Shang et al., 2010; Zhang et al., 2010; Wang et al., 2014). Among these methods, ELISA for GPV antibody detection is widely used by laboratory personnel due to its ability to simultaneously test large batches of samples, its ease of operation, and its convenience. Reported ELISA methods for detecting antibody against GPV infection mainly include ELISA that utilize VP3 full-length protein as the coating antigen (Zhang et al., 2010), ELISA that use VP3 antigen site VP3ep4–6 recombinant protein as the coating antigen (Tarasiuk et al., 2019), and ELISA that employ NS1 and VP3 fusion proteins for coating and can differentiate between natural infection and vaccine immunity (Zhang et al., 2020). These ELISA methods play a crucial role in the detection of antibody against GPV infection. Peptide-based ELISAs use artificially synthesized antigenic site polypeptides as coating antigens, avoiding the non-specificity issues caused by protein purified in prokaryotic expression systems. Currently, they have been applied in antibody detection for various diseases (Wang et al., 2002; Oleksiewicz et al., 2005; Tian et al., 2010; Dubois et al., 2012; Gao et al., 2012). Our research group has previously established a specific peptide-based ELISA method for detecting avian leukosis virus subgroup J, which exhibits excellent specificity and sensitivity (Qian et al., 2018).

This study involved the synthesis of a peptide derived from the antigenic region of the GPV VP3 protein for use in an ELISA-based antibody detection method. And the research stablished and evaluated this peptide-based ELISA method for detecting GPV antibodies. This method offers a more efficient and specific approach for clinical detection of GPV infection or assessment of vaccine immunity effectiveness.

## 2 Materials and methods

### 2.1 Virus, cells and serum

The goose plague cell-adapted virus (GPV-CZM) with GenBank accession number MT939904.1, serum from GPV-immunized mice, serum from non-immunized geese, HRP-labeled mouse anti-goose IgG (HRP-go3G11), and positive sera for Newcastle Disease Virus (NDV), H9 subtype avian influenza virus (AIV), avian reovirus (REO), Tembusu virus (TMUV), and goose astrovirus (GAstV) were all prepared and stored in our laboratory. Goose embryo fibroblasts (GEF) were prepared using 10-day-old non-immunized goose embryos. The low-virulence live vaccine for GPV (strain SYG41-50) was purchased from China National Pharmaceutical Group Yangzhou VAG Biological Engineering Co., Ltd. A total of 120 doses of immune goose serum and 50 doses of non-immune goose serum were generously donated by Suqian Lihua Animal Husbandry Co., Ltd.

### 2.2 Selection, alignment analysis, and synthesis of VP3 antigen peptide sequences

Using DNA Star software (Madison, Wisconsin, United States), the antigenicity of the GPV classical strain GB VP3 protein was predicted. Antigenic and hydrophilic amino acid sequences were selected for peptide synthesis. Additionally, the chosen peptide sequences were compared and analyzed against the various GPV vaccine strains.

All the peptides were synthesized by Shanghai Jietai Biotech Company. The crude peptides were purified in purity greater than 95%. The BSA conjugated polypeptide (BSA-VP3-1) was also synthesized and purified by Shanghai Jietai Biotech Company.

### 2.3 Procedure for peptide ELISA

BSA-VP3-1 peptide solution (1 μg/mL), diluted in 0.1 M carbonate/bicarbonate buffer (pH 9.6), was added to Maxisorp ELISA plates (NUNC, Thermo, Shanghai) at 100 μL per well and incubated at 4°C overnight. The following morning, each well was sealed with 360 μL of 5% skimmed milk in PBS solution and incubated at 37°C for 3 h. Afterward, the plates were washed three times with PBS containing 0.05% Tween and tapped dry on absorbent paper towels. Following the blocking and washing steps, serum samples to be tested, diluted at 1:200, were added and incubated at 37°C for 1 h. Washing was performed three times with PBS containing 0.05% Tween. Subsequently, 100 μL of a 1:1000 dilution of horseradish peroxidase-conjugated mouse anti-goose IgG (prepared in our laboratory) was added and incubated at 37°C for 1 h. After three washes, 100 μL of TMB substrate (Pierce, Thermo, United States) was added to each well and incubated at 37°C in the dark for 10 min. The reaction was terminated by adding 50 μL of 2 M sulfuric acid to each well. The absorbance at OD_450_nm was measured using a standard ELISA plate reader (Bio-TEK, Vermont, United States).

### 2.4 Testing the specificity of peptide-based ELSA

The established peptide-based ELISA antibody detection method was used for the cross-reactivity testing with the antibodies of Newcastle Disease Virus (NDV), H9 subtype avian influenza virus (AIV), avian reovirus (REO), Tembusu virus (TMUV), and goose astrovirus (GAstV). Non-immune goose serum served as the negative control.

### 2.5 Determination of repeatability and reproducibility

To assess precision, coefficients of variation (CV) for triplicates of eight samples were determined on the same plate to calculate intra-assay variability (within-plate variation). For reproducibility, CV (between-plate variation) was obtained by running triplicates of the same samples on two different plates using identical samples.

### 2.6 Indirect immunofluorescence assay

In a 96-well plate, approximately 4 × 10^4^ primary GEF cells were added to each well. After culturing in 5% growth medium for 1 day, the cells were infected with GPV and cultured in 1% maintenance medium for 4 days. The maintenance medium was then removed from all wells. Each well was fixed with 100 μL of acetone-ethanol fixative (−20°C pre-chilled) at room temperature for 5 min, followed by washing with PBS. The standard positive serum, negative serum, and the serum to be tested were diluted 50-fold in PBS. Two replicate wells were set up for each, and 100 μL of each diluted serum was added to the wells. The plate was incubated at 37°C for 1 h. After washing with PBS, each well was incubated with go-3G11 mouse anti-goose IgG (prepared in our laboratory at a concentration of 5 μg/mL at 37°C for 1 h). Following three washes with PBS, 100 μL of FITC-conjugated goat anti-mouse antibody (diluted 1:200) was added to each well and incubated at 37°C for 1 h. Afterward, the plate was washed five times with PBS, gently tapped dry, and 50 μL of a mixture containing 50% glycerol was added. The plate was then observed under a fluorescence microscope, and images were captured for recording.

### 2.7 Detection of dynamic changes in immune goose serum antibodies

One-day-old non-immunized goslings were purchased from Jiangsu Lihua Animal Husbandry Co., Ltd. They were divided into two groups: one group received commercial goose plague vaccine immunization (The low-virulence live vaccine (strain SYG41-50) was purchased from China National Pharmaceutical Group Yangzhou VAG Biological Engineering Co., Ltd.), and the other group served as a non-immunized control. Blood samples were collected from the immunized group on days 4, 8, 15, 22, 29, and 36 after immunization, and serum was separated. The dynamic changes in antibodies within the immunized geese were detected using the established peptide-based ELISA.

### 2.8 Comparison of the peptide-ELISA and agar gel precipitation test

120 serum samples from the Jiangsu Lihua Animal Husbandry goose breeding facility, which had been immunized against GPV, were sent for testing. A comparative experiment was conducted using both peptide-based ELISA and AGP methods to assess the positive detection rates of the two antibody detection methods.

## 3 Result

### 3.1 VP3 protein antigenicity region prediction analysis

**Figure 1:**
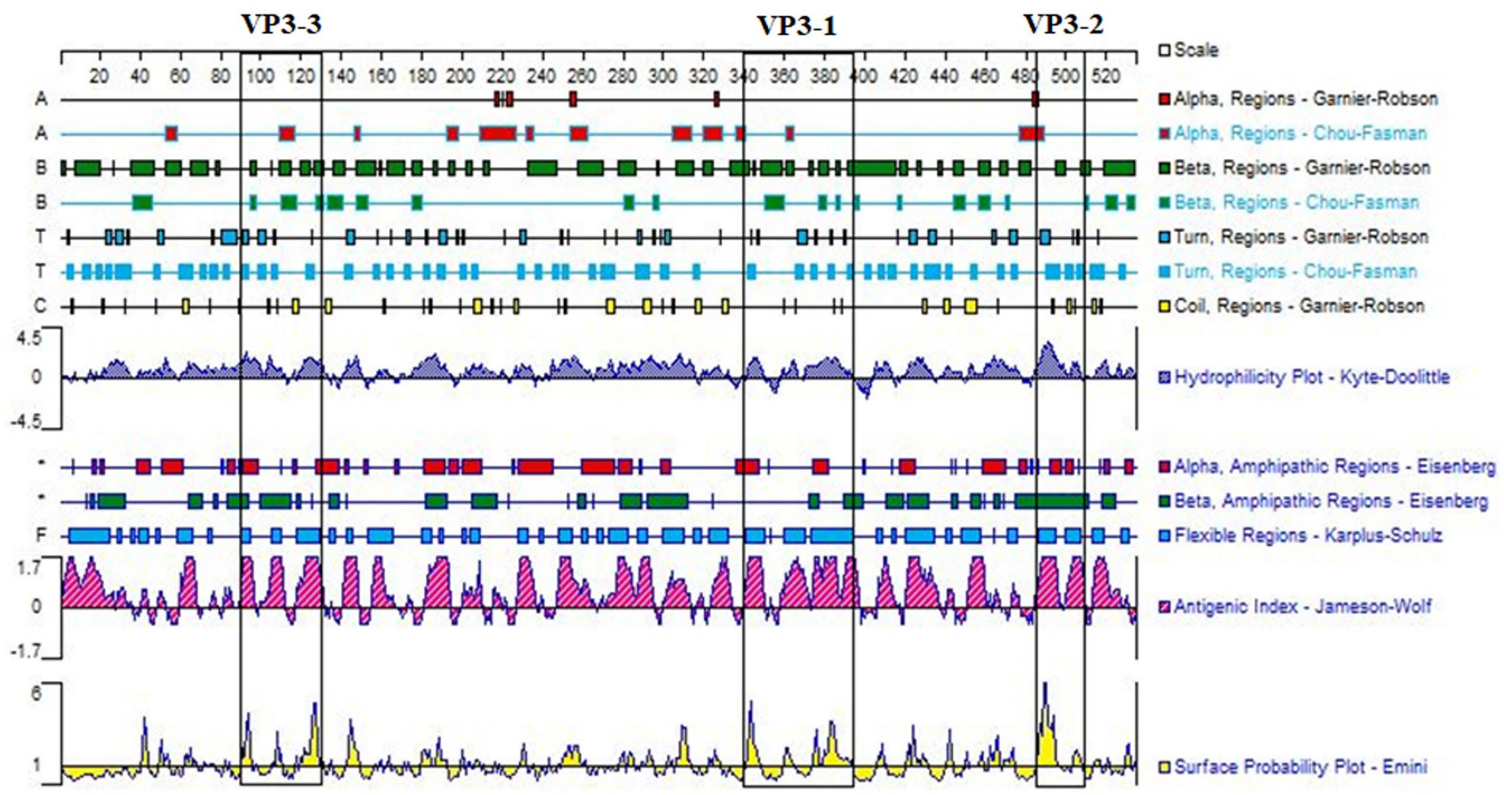
Antigenic domain analysis of GPV-VP3. Three peptide sequences, VP3-1 (358–392 aa), VP3-2 (485–509 aa), and VP3-3 (111–130 aa), framed in black, were selected based on the analysis of surface probability, antigenicity, hydrophilicity, and the secondary structure of the VP3 gene using DNAStar software. These sequences exhibited good antigenicity.

Using DNAStar software, comprehensive simulation analysis was performed on the surface properties such as surface probability, antigenicity, hydrophilicity, and secondary structure of the VP3 gene’s amino acid sequence (Figure 1). Ultimately, three peptide sequences with good antigenicity located on the surface were selected, namely VP3-1 (358-392aa), VP3-2 (485-509aa), and VP3-3 (111-130aa) (Table 1).

**Table 1:** Sequences of three selected polypeptides.

Peptide ID	Sequence	Location
VP3-1	VTDEQEVAPTNGVGWKPYGKTVTNEQNTTTAPTSS	358–392
VP3-2	LRKENSKRWNPEIQFTSNFSDRTSI	485–509
VP3-3	FKIFNVQVKEVTTQDQTKTI	111–130

The VP3-1 peptide is located in the 358–392 amino acids encoded by the VP3 of GPV; The VP3-2 peptide is located in the 485–509 amino acids encoded by the VP3 of the GPV virus; The VP3-3 peptide is located in the 111–130 amino acids encoded by the VP3 of GPV.

#### 3.1.1 Antigenic peptide sequence alignment analysis

**Figure 2:**
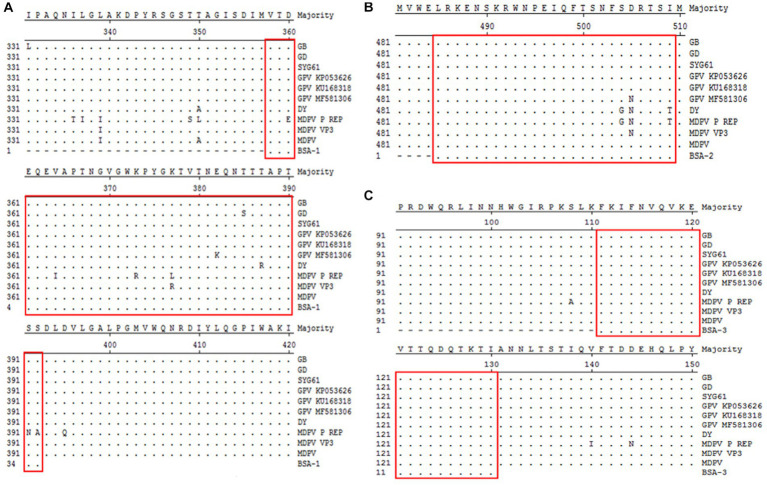
Comparative analysis of amino acid homology of polypeptides. The selected three peptide sequences framed in red were compared and analyzed against different goose parvoviruses. **(A)** Comparative analysis of amino acid homology of VP3-1(358–392 aa), **(B)** Comparative analysis of amino acid homology of VP3-2(485–509 aa), **(C)** Comparative analysis of amino acid homology of VP3-3(111–130 aa).

The selected three peptide sequences were compared and analyzed against different goose parvoviruses. VP3-1 showed homology ranging from 97.2 to 100% with goose parvovirus and from 83.3 to 100% with Muscovy duck parvovirus. VP3-2 exhibited homology ranging from 96.2 to 100% with goose parvovirus. VP3-3 showed 100% homology with all strains. The sequence alignment analysis results are shown in Figures 2A–C. Finally, the three amino acid sequences were sent to the company for synthesis as BSA-coupled peptides, namely BSA-VP3-1, BSA-VP3-2, and BSA-VP3-3, to be used as ELISA coating antigens.

### 3.2 Selection of the optimal coating peptide antigen for ELISA

BSA-VP3-1, BSA-VP3-2, and BSA-VP3-3 peptides were individually or combined as coating antigens at a concentration of 1 μg/mL. A known GPV positive serum at a 1:100 dilution was used as the primary antibody, and an HRP-labeled mouse anti-goose IgG (HRP-go3G11) at a 1:200 dilution was used as the secondary antibody for the reaction. Based on the OD_450_ reading results, it was found that BSA-VP3-1 peptide coating individually yielded the best results, with the highest P/N value (results are shown in Table 2). Subsequent experiments will determine BSA-VP3-1 peptide (1 μg/mL) as the coating antigen for GPV antibody detection in peptide ELISA.

**Table 2:** Peptide ELISA reaction with positive goose serum.

Peptides	1	2	3	1 + 2	1 + 3	2 + 3	1 + 2 + 3
Positive Serum	0.862	0.434	0.366	0.957	0.768	0.654	0.791
	0.844	0.433	0.395	0.950	0.769	0.633	0.780
Negative Serum	0.139	0.260	0.110	0.319	0.120	0.369	0.172
	0.145	0.255	0.117	0.354	0.123	0.346	0.158
PBST	0.076	0.068	0.058	0.087	0.086	0.093	0.085

The number 1, 2, 3 mean ELISA coated with peptide VP3-1, VP3-2 and VP3-3, respectively.

### 3.3 Development of BAS-VP3-1 peptide ELISA for detecting GPV Antibodies

The optimal dilution of the coating antigen, serum and HRP conjugated mouse anti goose IgG in the peptide ELISA were determined using a checkerboard titration. The optimal dilution of reagents was determined at 1 μg/mL for BSA-VP3-1 peptide, 5% skimmed milk in PBS with 0.05% Tween 20 for blocking reagent, 1:100 for serum sample and 1:400 for the secondary horseradish peroxidase-conjugated mouse anti-goose immunoglobulin. The 100 μL of 3,30,5,50-tetramethylbenzidine (TMB) was used for exposure at 37°C for 15 min away from light. The reaction was stopped by the 50 μL of 2 M H_2_SO_4_. Optical density (OD) values were measured at 450 nm. Using these conditions, the best signal with minimum background was obtained in the peptide-ELISA.

### 3.4 Determination of the cut-off value

Using the optimized ELISA method established in the above experiments, 50 non-immune goose sera were tested, and their OD_450_nm values were determined. The average values of the duplicate wells were calculated, and the results are summarized in Table 3. Among them, well E7 serves as the positive control, and well E8 serves as the blank control. Using SPSS software for analysis, the average value (x̄) is 0.219, the standard deviation (SD) is 0.064. The detection cutoff for positive samples (Cp) is calculated as (x̄) + 3(SD), which equals 0.219 + 3 × (0.064) = 0.411. The upper limit for negative samples (Cn) is calculated as (x̄) + 2 × (SD), which equals 0.219 + 2 × (0.064) = 0.347. Therefore, an OD_450_ value of ≤0.347 is considered negative, an OD_450_ value of ≥0.411 is considered positive, and values in between are considered suspicious.

**Table 3:** Determination of the cut-off value in ELISA assay.

	1	2	3	4	5	6	7	8	9	10	11
A	0.233	0.218	0.201	0.311	0.310	0.164	0.143	0.295	0.236	0.350	0.320
B	0.153	0.224	0.311	0.195	0.178	0.249	0.182	0.262	0.202	0.168	0.152
C	0.277	0.153	0.169	0.155	0.148	0.198	0.171	0.124	0.225	0.350	0.323
D	0.285	0.240	0.190	0.130	0.290	0.248	0.180	0.180	0.187	0.307	0.183
E	0.148	0.307	0.153	0.158	0.248	0.180	1.851	0.082			

### 3.5 The cross-reaction test of the peptide ELISA

**Figure 3:**
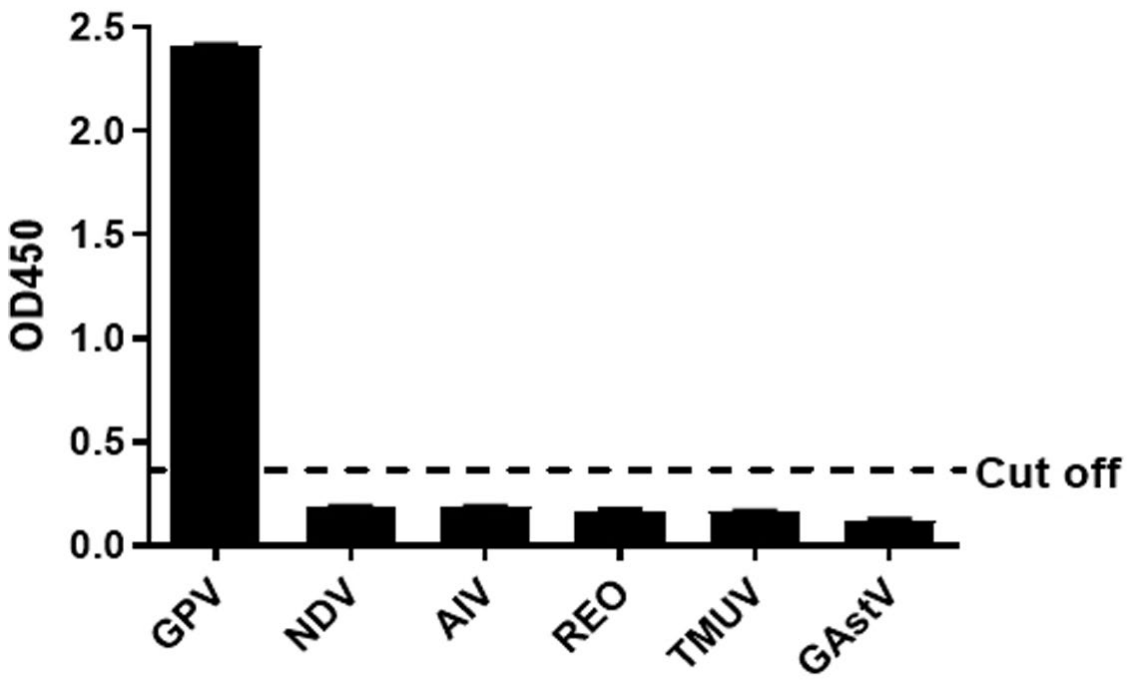
Cross reaction test. The BSA-VP3-1 peptide was used as the coating antigen and positive goose serum against NDV, H9 AIV, REO, TMUV, and GAstV were tested. All OD_450_ values except that of GPV were lower than the cut-off value. The bars in figure mean the average OD_450_ value of triplicate experiments. NDV: Newcastle Disease Virus, AIV: H9 subtype avian influenza virus, REO: avian reovirus, TMUV: Tembusu virus, GAstV: goose astrovirus.

The other serums of goose pathogens including Newcastle virus (NDV), H9 avian influenza virus (H9 AIV), reovirus (REO), Tembusu virus (TMUV), and Goose Astrovirus(GAstV)were used for the specificity evaluation of the peptide ELISA. The result in the Figure 3 showed that no cross-reactions were detected by the peptide ELISA with the OD values ranging from 0.093 to 0.202 (Figure 3), which demonstrating a good specificity of the BSA-VP3-ELISA for detection of the GPV antibody.

### 3.6 Repeatability and reproducibility of the VP3 peptide-ELISA

The repeatability and reproducibility of the peptide ELISA was determined by eight sera samples. The intraplate variation showed CVs from 3.12 to 6.62%, whereas the inter-plate variation showed CVs from 4.45 to 6.92%. The minor variation in the results showed that the VP3 peptide ELISA was reproducible.

8. The dynamic antibodies in immune goose were detected by peptide ELISA.

**Figure 4:**
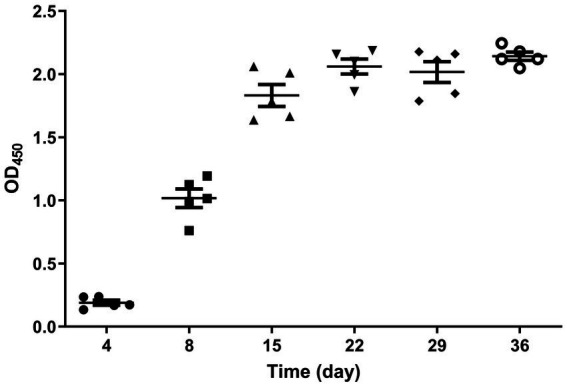
Dynamic diagram of serum antibody changes in immunized geese. The serum samples collected from the immunized group on days 4, 8, 15, 22, 29, and 36 after immunization were tested using the established peptide-based ELISA. The immunization success rate was 100%, and the antibody levels reached their peak at 22 days post-immunization.

The results of the peptide ELISA detection showed that on the 4th day post-immunization, no antibodies were produced. By the 8th day, a high level of antibodies had already developed, and as time passed, the antibody levels in the body reached their peak at 22 days. The immunization success rate was 100%, and individuals of the same age showed minimal fluctuations in antibody levels (Figure 4). The non-immunized control group consistently tested negative, but the results are not shown.

### 3.7 Comparison of VP3 peptide-ELISA and AGP test

**Figure 5:**
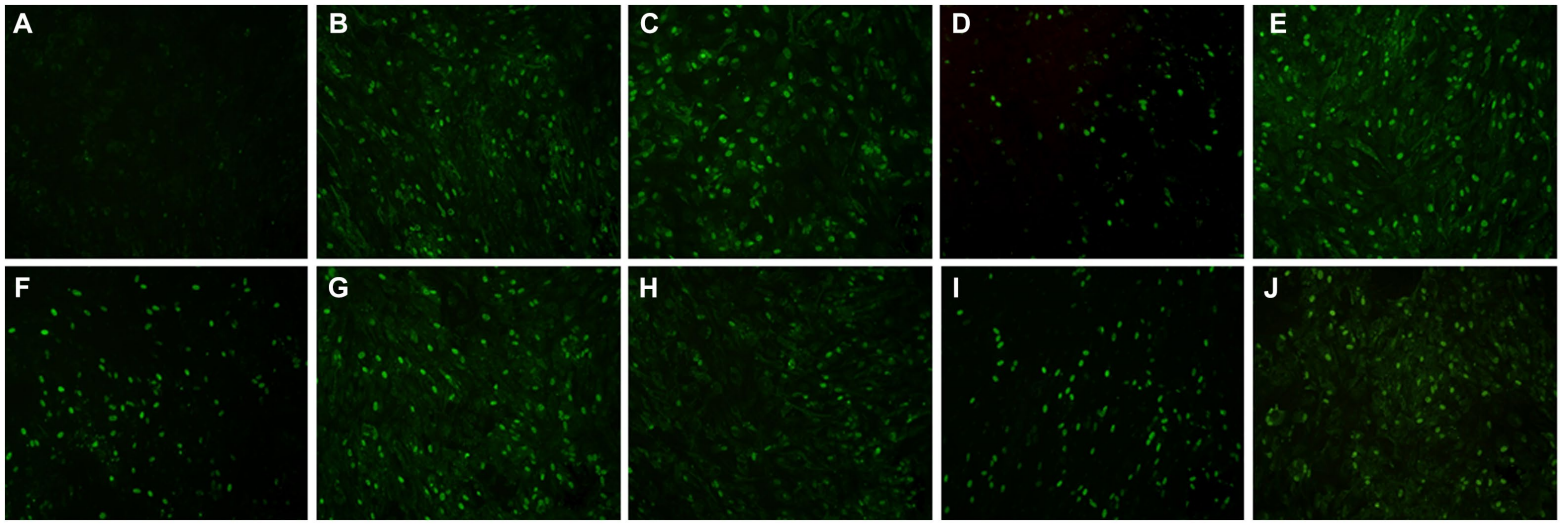
The result of nine serum samples negative in the AGP evaluated with IFA. To further verify the specificity of nine serum samples that tested negative in the AGP assay, we conducted an IFA using these sera as primary antibodies. Panel **(A)** served as the negative control, while panels **(B–J)** displayed positive results from serum samples that were negative in AGP but positive in VP3 peptide-ELISA.

A total of 120 goose sera were examined in parallel comparing the VP3 peptide-ELISA and AGP test. All the sera samples were positive using the VP3 peptide-ELISA and 9 were negative in the AGP test. The positivity of peptide-ELISA was 100% (120/120), as compared with 84.17% (101/120) for AGP test (Table 4). To further verify the specificity of 9 sera samples which were negative in AGP test, the IFA was carried out with these sera as primary antibodies. The results in Figure 5 showed all the 9 sera samples were positive in IFA assay, which indicated that the VP3 peptide-ELISA was more sensitive than AGP test.

**Table 4:** Detection of anti-GPV antibodies in clinical samples.

		peptide-ELISA		AGP	
		Positive	Negative	Positive	Negative
IFA	Positive	120	0	101	9
	Negative	0	0	0	0

## 4 Discussion

Infection with GPV in 1 to 20-day-old goslings or ducklings can lead to a highly contagious, transmissible, highly pathogenic, and high mortality acute or subacute septicemic infectious disease in animals. The mortality rate can be extremely high, even reaching 100% (Calnek, 1991; Swayne, 2020). The susceptibility of waterfowl can lead to large-scale outbreaks on poultry farms through direct or indirect transmission routes, posing a serious threat to the development of the poultry industry. Currently, goose farms primarily rely on hyperimmune serum and vaccine immunization to protect against viral infections in poultry production (Swayne, 2020). One of the more effective and cost-efficient methods is to induce the production of antibodies in breeder geese. Gosling can then passively acquire maternal antibodies through the egg yolk, providing them with passive immunity (Fang, 1962; Kisary and Derzsy, 1974). When administering booster vaccinations to goslings in the later stages of their development, it’s essential to conduct antibody testing in advance. This is because the presence of maternal antibodies in their system can impact the effectiveness of the vaccine-induced immunity (Fan et al., 2013; Zhang et al., 2020). Currently, in clinical practice, GPV antibody detection methods primarily involve agar gel diffusion tests and neutralization assays. However, agar gel diffusion tests require the concentration of antigens and high-titer goose hyperimmune serum, and they may have a low positive detection rate (Kisary and Derzsy, 1974; Swayne, 2020). Neutralization assays have higher sensitivity and specificity, but the assay itself is labor-intensive, time-consuming, and challenging to perform in large-scale testing (Kisary and Derzsy, 1974). Furthermore, ELISA antibody detection methods utilizing proteins expressed in prokaryotic systems have also been reported (Crabb and Studdert, 1995; De Diego et al., 1997; Johne et al., 2004). However, these methods can be susceptible to non-specific interference from the prokaryotically expressed proteins. Hence, there is a need in clinical production for a high-throughput and more specific method to detect GPV antibodies. In this study, we used bioinformatics software to analyze and predict the antigenic regions of the GPV main protective antigen VP3. We then established a specific method for detecting goose parvovirus antibodies by coupling it with BSA for use as an ELISA coating antigen. The results demonstrated strong specificity, good stability, and high accuracy in antibody detection. The experimental results comparing the VP3 peptide ELISA method with the traditional AGP method indicate that the peptide ELISA method exhibits better sensitivity. In the monitoring of immune dynamics in goslings vaccinated with GPV, the VP3 peptide ELISA method is also effective in detecting the dynamic changes in antibodies in the serum of immunized goslings. These results suggest that the peptide ELISA method demonstrates advantages in high throughput and sensitivity. Furthermore, the high homology observed in the VP3 sequences of GPV, MDPV, and Novel GPV (NGPV) suggests that this peptide ELISA method might be applicable for detecting antibodies against MDPV and NGPV as well. However, further validation with a larger number of clinical serum samples would be necessary to confirm its effectiveness for these viruses. Moreover, this study focuses on detecting the levels of GPV antibodies in the geese. At present, this peptide ELISA cannot differentiate the antibodies from the natural infection and vaccination. The peptide-based ELISA used to differentiate needs further investigation in the future.

In conclusion, the experimental results of this study collectively demonstrate that the VP3 peptide ELISA detection method established here offers a high-throughput, highly sensitive, and specific approach for antibody detection, requiring only a small amount of serum. The method developed for detecting goose parvovirus antibodies in this research provides a novel method for clinical detection and prevention of goose plague. Further optimization and refinement through additional sample testing are still needed to enhance its performance.

## Data availability statement

The original contributions presented in the study are included in the article/supplementary material, further inquiries can be directed to the corresponding authors.

## Ethics statement

The animal study was approved by Animal Care Committee of Yangzhou University in China. The study was conducted in accordance with the local legislation and institutional requirements.

## Author contributions

DQ: Data curation, Formal analysis, Methodology, Software, Writing – original draft. LW: Data curation, Methodology, Software, Validation, Writing – original draft. CG: Data curation, Formal analysis, Methodology, Software, Validation, Writing – original draft. HS: Data curation, Methodology, Validation, Writing – review & editing. YY: Investigation, Validation, Writing – review & editing. AQ: Project administration, Supervision, Writing – review & editing. AH: Investigation, Project administration, Supervision, Writing – review & editing. KQ: Data curation, Formal analysis, Investigation, Methodology, Project administration, Software, Supervision, Validation, Writing – review & editing.
